# Prediction of Posterior-to-Anterior Corneal Curvature Radii Ratio in Myopic Patients after LASIK, SMILE, and PRK Using Multivariate Regression Analysis

**DOI:** 10.3390/jcm12134536

**Published:** 2023-07-07

**Authors:** David S. Cha, Majid Moshirfar, Michael S. Herron, Jordan M. Santos, Phillip C. Hoopes

**Affiliations:** 1 School of Medicine, Saint Louis University, Saint Louis, MO 63104, USA; chads@slu.edu; 2 Hoopes Vision Research Center, Hoopes Vision, Draper, UT 84020, USA; pch@hoopesvision.com; 3 John A. Moran Eye Center, University of Utah School of Medicine, Salt Lake City, UT 84132, USA; 4 Utah Lions Eye Bank, Murray, UT 84107, USA; 5 University of Nevada, Reno School of Medicine, Reno, NV 89557, USA; michaelherron@med.unr.edu; 6 University of Arizona College of Medicine Phoenix, Phoenix, AZ 85004, USA; jordansantos@arizona.edu

**Keywords:** cornea, refractive surgery, keratometry, asphericity, posterior-to-anterior corneal radii ratio

## Abstract

The ratio of posterior-to-anterior curvature radii of the cornea (P/A ratio) is an important element in determining corneal refractive power. P/A ratio has been well studied in patients prior to undergoing refractive surgery, but its postoperative value remains less so. We aimed to examine the value of preoperative characteristics of refractive surgery patients in predicting the 1-year postoperative P/A ratio in LASIK, PRK, and SMILE using both linear and multivariate regression analyses. This was a retrospective study that included patients with manifest refraction spherical equivalents (MRSE) from −7.71D to −0.25D. In total, 164 eyes underwent LASIK, 183 underwent PRK, and 46 underwent SMILE. All patients had preoperative and 1-year postoperative front sagittal and back sagittal keratometry measurements at 4, 5, and 6 mm around the corneal vertex. Postoperative P/A after LASIK, PRK, and SMILE was found to be significantly correlated with MRSE and preoperative P/A. Stepwise variable selection in multivariate regression revealed that spherical equivalent was the most significant predictor of postoperative P/A. When coupled with other preoperative characteristics, including P/A, age, asphericity, and keratometry, the multivariate regressions were able to produce models with high predictive value in LASIK (adjusted R^2^: 0.957), PRK (adjusted R^2^: 0.934), and SMILE (adjusted R^2^: 0.894).

## 1. Introduction

Corneal refractive power is vital to assess outcomes in refractive and cataract surgery. It depends on several factors, including corneal thickness, tissue index refraction, and the curvature of the anterior and posterior corneal surface. In the past, optical devices such as keratometers could only measure anterior corneal curvature and thickness. Calculation of corneal refractive power was therefore based on fixed parameters such as an estimated posterior-to-anterior corneal curvature radii ratio (P/A ratio) of 0.82. However, the arrival of optical coherence tomography and Scheimpflug imaging allow for simultaneous measurement of the anterior and posterior corneal curvature, resulting in an accurate measurement of the P/A ratio. Inaccuracies in not only corneal refractive power but also P/A ratio have been shown to contribute significantly to the error in intraocular lens calculations [1]. An accurate prediction of P/A ratio could therefore substantially improve outcomes in cataract surgery.

Additionally, the P/A ratio could be a useful indicator of the risk of keratectasia. P/A, by nature of its quotient, can quantify the changes in posterior corneal curvature relative to the anterior surface when measured before and after a corneal intervention. In the same vein, posterior corneal elevation commonly occurs after refractive surgery and represents the earliest signs of iatrogenic keratectasia [2]. Expanding the metrics by which we evaluate changes of the posterior surface could allow for improved diagnosis of keratectasia and related conditions.

Many studies have characterized P/A ratio in patients who have not yet undergone refractive surgery, but few have evaluated it postoperatively. We aimed to describe P/A ratio in patients after undergoing refractive surgery and hypothesized that certain preoperative characteristics could correlate with its value. The purpose of this study was to first examine demographic factors and preoperative measurements that could influence P/A ratio. Additionally, we conducted multivariate analyses not only to assess which of these preoperative variables contributed the most predictive value but also to construct a model by which to accurately predict the postoperative P/A ratio in LASIK, PRK, and SMILE.

## 2. Materials and Methods

### 2.1. Study Setting

The present study was conducted at a corneal refractive surgery center with approval by the Biomedical Research Alliance of the New York Institutional Review Board (#A20-12-547-823). All participating patients provided informed consent. A group of patients diagnosed with myopia or compound myopic astigmatism received either PRK, LASIK, or SMILE between June 2020 to March 2022. Criteria for inclusion were the following: adults ≥ 18 years old and best corrected visual acuity (BCVA) 20/25 or better. Exclusion criteria included the following: ocular disease (retinal detachment, macular degeneration, keratoconus, glaucoma, cataract formation), infectious or autoimmune disease (herpes simplex infection, systemic lupus erythematosus), hyperopic astigmatism, hyperopia, eyes with monovision, and patients requiring subsequent enhancement refractive surgery. The group of patients was divided into 3 separate groups per type of surgery received including PRK, LASIK, and SMILE.

### 2.2. Data Collection

All patients included in the study were evaluated preoperatively with manifest refraction, visual acuity exam, tonometry, slit-lamp examination, and dilated fundus exam. These were repeated at the 1-year postoperative visit. All patients also received tomographic imaging via Pentacam (Oculus) both pre- and postoperatively. Tomography data were exported into Microsoft Excel. Keratometry was measured at a 3-mm zone in 15-degree intervals centered around the corneal vertex of the anterior cornea as the average of the flat (K1) and steep (K2) simulated keratometry readings. Keratometry was also measured at 4-, 5-, and 6-mm zones around corneal vertex for the anterior and posterior cornea. Anterior and posterior corneal radii were calculated using the equations K_F_ = (1.3376 − 1)/r × 1000 and K_B_ = (1.336 − 1.376)/r × 1000, respectively, where r is the corneal radius, K_F_ is anterior keratometry, and K_B_ is posterior keratometry. Corneal asphericity (Q value) was measured at an 8-mm zone around the vertex.

A single surgeon (MM) performed all surgeries at one site. PRK and LASIK surgical equipment included the WaveLight Allegretto Wave Excimer Laser System (Alcon Laboratories, Fort Worth, TX, USA). In LASIK, a 100 µm flap was performed by an FS200 (Alcon Laboratories, Fort Worth, TX, USA) femtosecond laser. The planned optical zone and blended zones were created at 6.5 mm and 9 mm, respectively, for LASIK and PRK patients. SMILE was conducted with Visumax 500 kHz femtosecond laser (Carl Zeiss Meditec, Jena, Germany) with the creation of a 6.5 mm optical zone and a 0.5 mm transition zone for all SMILE patients. Specific details of the surgical procedures can be found in the following references [3,4,5].

### 2.3. Statistics

Statistical analyses were conducted using Microsoft Excel and IBM SPSS Statistical software (ver. 29.0; SPSS Inc., Chicago, IL, USA). The Shapiro–Wilk test was used to assess normalcy of data distribution. After populations were determined to be non-normal in distribution, Spearman’s rank correlation was run for analysis of the effect on independent preoperative characteristics on the postoperative P/A ratio. Preoperative characteristics that were found to be significant were used for multivariate regression. Collinearity was further assessed with the variance inflation factor and collinearity tolerance statistics. Variables were excluded if the variance inflation factor exceeded 4 or if tolerance was below 0.25. Residual plots were produced between the standardized residual error and the predicted value for postoperative P/A for LASIK, PRK, and SMILE at 6 mm. The independence of residuals was assessed using Durbin–Watson tests, with values of 1.5 to 2.5 indicating independence. For an alpha value of 0.05 and a power of 0.80, a minimum sample size of 35 eyes was calculated for a medium effect size using G*Power (version 3.1, Franz Faul, Unversität Kiel, Germany). Significance was adjusted using Bonferroni corrections.

## 3. Results

### 3.1. Population Characteristics

This study included a total of 393 eyes from 215 patients that underwent LASIK, SMILE, or PRK surgery, consisting of 99 females (46.0%) and 116 males (54.0%). Of these eyes, 164 (91 patients) underwent LASIK, 183 (100 patients) underwent PRK, and 46 (25 patients) underwent SMILE. Demographic data of the eyes are shown in Table 1.

### 3.2. Linear Regression Analysis

Preoperative characteristics and their correlation with P/A both preoperatively and postoperatively are shown in Table 2. In all patients preoperatively, P/A ratio was found to positively correlate with age (4 mm: r = 0.195, 5 mm: r = 0.213, 6 mm: r = 0.239), posterior Q (4 mm: r = 0.400, 5 mm: r = 0.376, 6 mm: r = 0.336), and posterior Km (4 mm: r = 0.602, 5 mm: r = 0.580, 6 mm: r = 0.556). P/A negatively correlated with MRSE (4 mm: r = −0.115, 5 mm: r = −0.106, 6 mm: r = −0.106), anterior Km (4 mm: r = −0.105), and pachymetry (4 mm: r = −0.450, 5 mm: r = −0.440, 6 mm: r = −0.431).

**Figure A1:**
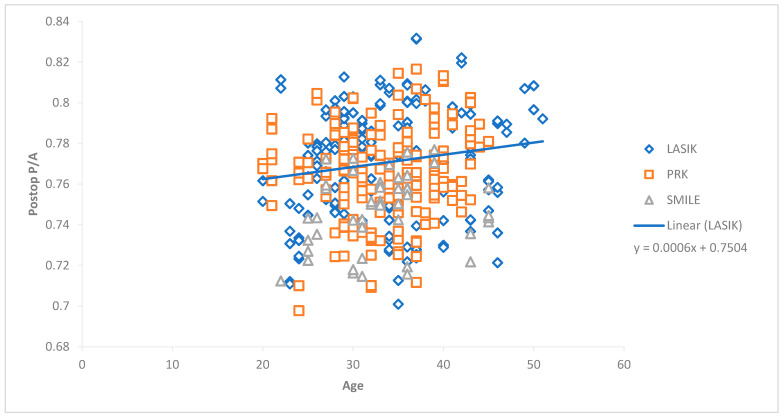
Scattergram showing correlation between age and postoperative P/A at 6-mm zone after LASIK, SMILE, and PRK.

**Figure A2:**
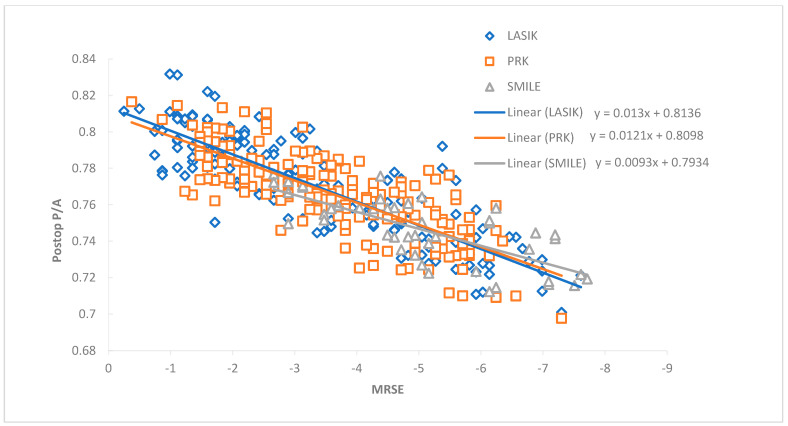
Scattergram showing correlation between MRSE and postoperative P/A at 6-mm zone after LASIK, SMILE, and PRK.

**Figure A3:**
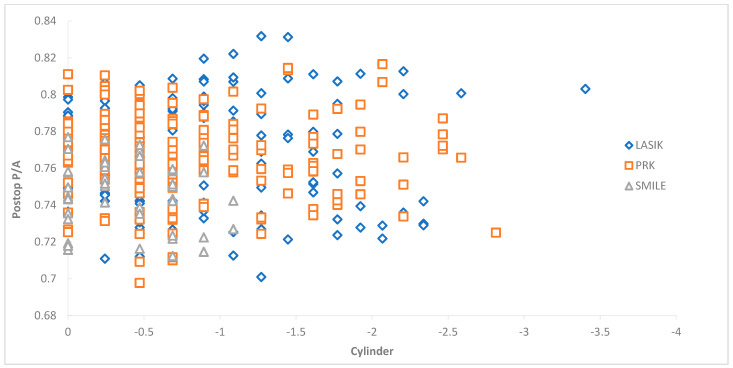
Scattergram between preoperative cylinder and postoperative P/A at 6-mm zone after LASIK, SMILE, and PRK.

**Figure A4:**
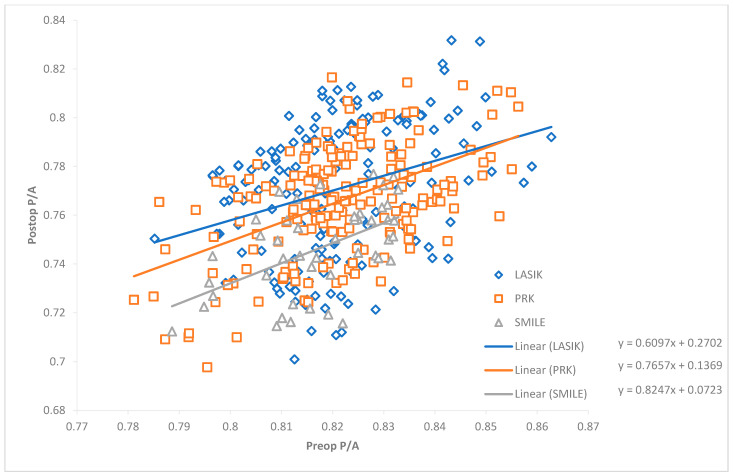
Scattergram showing correlation between preoperative and postoperative P/A at 6-mm zone after LASIK, SMILE, and PRK.

When looking at postoperative PA, age was found to positively correlate with P/A at the 6-mm zone in LASIK patients (r = 0.153, Table 2 and Figure A1). MRSE was found to positively correlate with P/A at 4-mm, 5-mm, and 6-mm zones in LASIK (r = 0.833, 0.837, 0.825), PRK (r = 0.791, 0.799, 0.779), and SMILE (r = 0.747, 0.772, 0.749) (Table 2 and Figure A2). Sex and cylinder were not correlated with postoperative P/A at any zone within any surgery group (Table 2 and Figure A3). Preoperative P/A also positively correlated at all zones and all surgeries (LASIK r = 0.289, 0.298, 0.324, PRK r = 0.388, 0.391, 0.410, SMILE r = 0.573, 0.536, 0.503, Table 2 and Figure A4).

**Figure A5:**
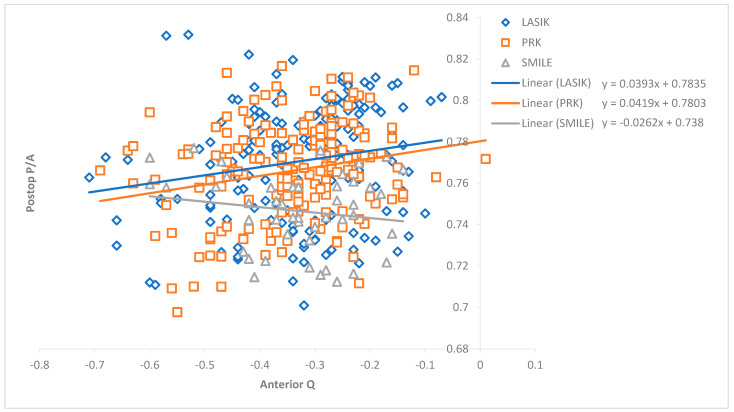
Scattergram showing correlation between anterior asphericity (Q) and postoperative P/A at 6-mm zone after LASIK, SMILE, and PRK.

**Figure A6:**
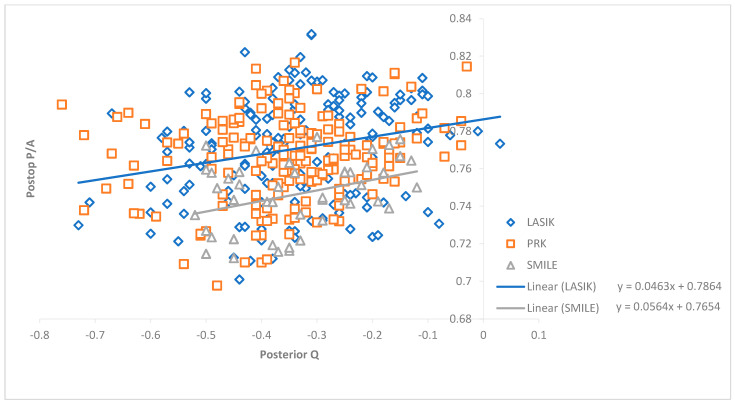
Scattergram showing correlation between preoperative posterior asphericity (Q) and postoperative P/A at 6-mm zone after LASIK, SMILE, and PRK.

**Figure A7:**
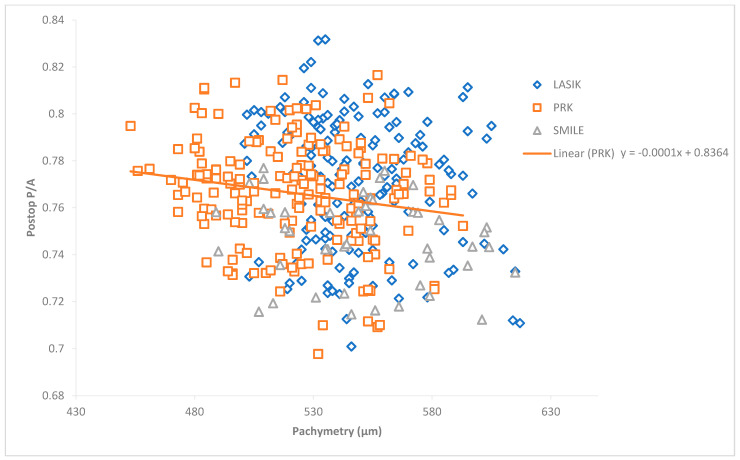
Scattergram showing correlation between preoperative pachymetry and postoperative P/A at 6-mm zone after LASIK, SMILE, and PRK.

Anterior Q value was found to positively correlate at 4-mm, 5-mm, and 6-mm zones in LASIK (r = 0.200, 0.194, 0.197) and PRK (r = 0.180, 0.183, 0.186) (Table 2 and Figure A5). Anterior Q value was not correlated with postoperative P/A in the SMILE group at any zone. Posterior Q value was positively correlated with postoperative P/A in all zones in LASIK (4 mm: r = 0.277, 5 mm: r = 0.261, 6 mm: r = 0.251) and SMILE (4 mm: r = 0.406, 5 mm: r = 0.374, 6 mm: r = 0.351) (Table 2 and Figure A6). However, it was only positively correlated in PRK at 4 mm (r = 0.177) and 5 mm (r = 0.155). Pachymetry was found to negatively correlate with postoperative P/A at 4-mm (r = −0.167), 5-mm (r = −0.161), and 6-mm (r = −0.160) zones in PRK, but not in SMILE or LASIK (Table 2 and Figure A7).

Anterior keratometry positively correlated with P/A ratio in LASIK at 4 mm (r = 0.226), 5 mm (r = 0.224), and 6 mm (r = 0.240), but negatively correlated with P/A ratio in PRK (r= −0.225, −0.197, −0.194). Posterior keratometry was found to positively correlate with postop P/A at all zones in PRK (4 mm: r = 0.381, 5 mm: r = 0.369, 6 mm: r = 0.379) and SMILE (4 mm: r = 0.440, 5 mm: r = 0.396, 6 mm: r = 0.392) patients. Posterior keratometry was not found to be correlated with P/A in LASIK patients (Table 2).

### 3.3. Multivariate Regression Analysis

All of the independent variables listed above were also run in multivariate analyses to examine their effect on postoperative P/A ratio in LASIK patients at 4-mm, 5-mm, and 6-mm central zones in Table 3. At 4 mm, MRSE, preoperative P/A ratio, anterior corneal radius, age, and anterior corneal asphericity were selected as independent variables to predict postoperative P/A ratio (R^2^: 0.946, *p* < 0.001). At 5 mm, MRSE, preoperative P/A ratio, age, and posterior corneal radius were selected as predictors of postoperative P/A ratio (R^2^: 0.943, *p* < 0.001). At 6 mm, MRSE, preoperative P/A ratio, age, anterior corneal radius, and anterior corneal asphericity were selected as predictors of postoperative P/A ratio (R^2^: 0.957, *p* < 0.001).

Multivariate regression was also performed for PRK patients in Table 4. At 4 mm, MRSE, preoperative PA, anterior Q value, and pachymetry were all selected as independent variables as predictors of postoperative P/A ratio (R^2^: 0.900, *p* < 0.001). In addition to the previous independent variables, age was selected as a preoperative predictor of postoperative P/A in step-down multivariate analysis at both 5 mm (R^2^: 0.930, *p* < 0.001) and 6 mm (R^2^: 0.936, *p* < 0.001).

Finally, multivariate regression was also repeated for SMILE patients in Table 5. At 4 mm, MRSE, preoperative PA, and posterior Q values were selected as independent variables as predictors of postoperative P/A (R^2^: 0.882, *p* < 0.001). In addition to the previous preoperative independent variables, age was also selected as a preoperative predictor of postoperative P/A in step-down multivariate analysis at both 5 mm (R^2^: 0.900, *p* < 0.001) and 6 mm (R^2^: 0.904, *p* < 0.001).

**Figure A8:**
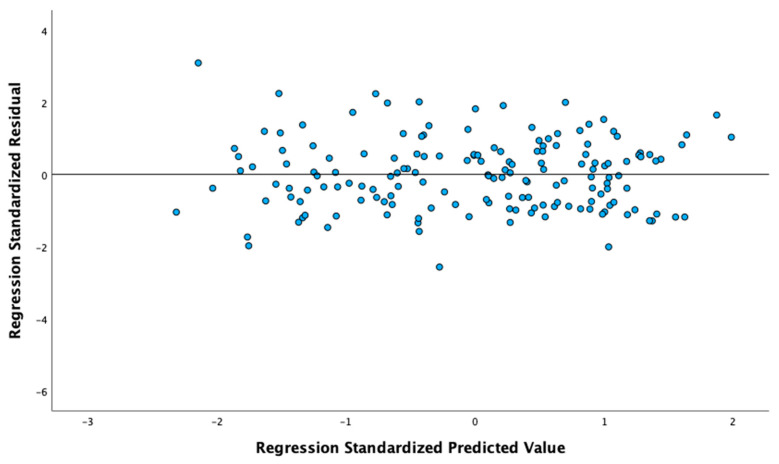
Residual plots of multivariate regression analyses of postoperative P/A in LASIK patients at 6-mm zone.

**Figure A9:**
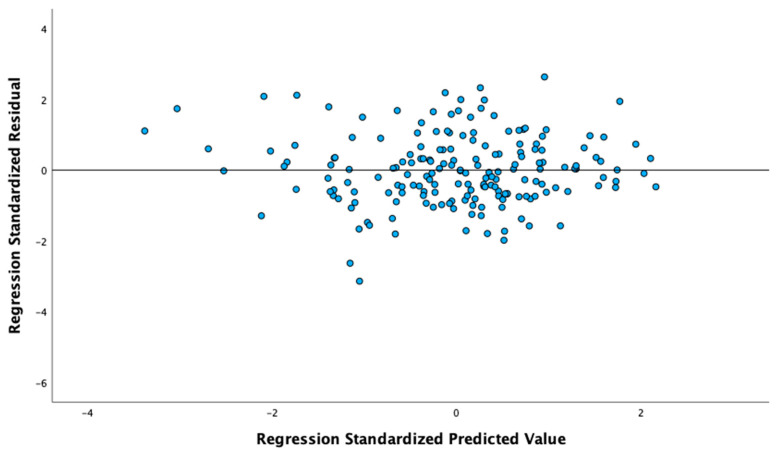
Residual plots of multivariate regression analyses of postoperative P/A in PRK patients at 6-mm zone.

**Figure A10:**
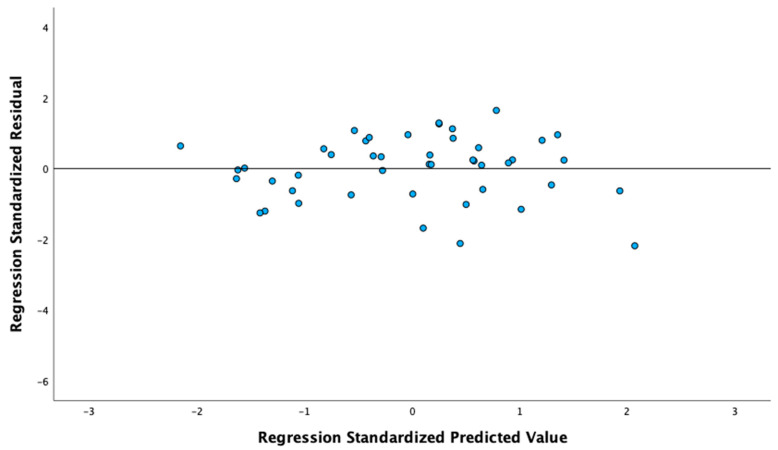
Residual plots of multivariate regression analyses of postoperative P/A in SMILE patients at 6-mm zone.

Residual plots are shown for postoperative P/A at 4 mm, 5 mm, and 6 mm for LASIK, PRK, and SMILE (Figure A8, Figure A9 and Figure A10). All residual plots demonstrate a random distribution pattern. The observations were independent in all 9 multivariate analyses (Durbin–Watson: LASIK 4 mm = 2.024, 5 mm = 1.996, 6 mm =1.921, PRK 4 mm = 1.857, 5 mm = 1.878, 6 mm = 1.921, SMILE 4 mm = 1.920, 5 mm = 2.051, 6 mm = 2.173).

## 4. Discussion

Our study looked at three populations that had similar mean ages and an even distribution of males and females. However, the populations have different average cylinder value, spherical equivalent, and pachymetry. This is largely due to certain characteristics being optimal for specific surgeries. For instance, SMILE is optimal for severely myopic patients but can only correct up to 3 diopters of cylinder. PRK is more suited to those patients with thinner corneas. Some may say that these differences could hinder the strength of our study, but it is critical to note that we never directly compare the surgery groups against one another and are simply studying the correlations within each group itself.

In our preoperative combined population, age was found to positively correlate with P/A ratio, suggesting a relative steepening of the anterior cornea in older patients. This finding is reflected in a study by Hasegawa et al. that also found a positive correlation, but it contradicts a study by Dubbelman that instead found a negative one [6,7]. This discrepancy could be explained by the fact that Dubbelman looked at the P/A ratio of asphericity, whereas Hasegawa and our study looked at the P/A ratio of radii. Postoperatively, age was not correlated with P/A ratio after all surgeries at all diameters except for LASIK at 6 mm. This could suggest that after LASIK, PRK, and SMILE, age has a relatively proportionate effect on the anterior and posterior cornea.

Spherical equivalent had a negative correlation with preoperative P/A in the combined population in our study. Although Tang et al. found no significant correlation between the two, they demonstrated a slight negative trend, which is consistent with our study [8]. This suggests that patients who are more myopic have a greater preoperative PA ratio. Interestingly, spherical equivalent had a positive correlation with postoperative P/A at 4 mm, 5 mm, and 6 mm across all surgical groups. This suggests that patients who are more myopic preoperatively will have a lower P/A ratio postoperatively in LASIK, PRK, or SMILE. This finding is expected as the P/A ratio is lower due to the increased anterior radius length that occurs in refractive surgery of myopic patients.

Preoperative P/A positively correlated with postoperative P/A globally across all zones and surgery groups. This indicates that patients with higher preoperative P/A ratios can expect to have a higher postoperative P/A ratio.

When we looked at all patients preoperatively, we found no correlation with anterior Q value (Table 2). This is consistent with what is found in the literature where no association between anterior asphericity and preoperative P/A ratio was reported [8]. Interestingly, anterior Q value positively correlated with P/A postoperatively in LASIK and PRK patient at all zones. This suggests that as the anterior surface becomes more oblate, P/A ratio increases in LASIK and PRK. This correlation was not found in SMILE. This discrepancy could be explained by the fact that SMILE is a delicate procedure that preserves the corneal epithelium and Bowman’s layer and thus has a relatively mild disturbance of the anterior surface compared to PRK and LASIK.

Posterior Q value was positively correlated with preoperative P/A at all zones, which is consistent with the findings of the Tang study. We found that posterior Q continued to positively correlate with postoperative P/A in LASIK and SMILE at 4 mm, 5 mm, and 6 mm as well as in PRK at 4 mm and 5 mm. This suggests that corneas that are more oblate will have a greater P/A ratio compared to less oblate corneas after LASIK, PRK, or SMILE.

Pachymetry was negatively correlated with preoperative P/A, which is consistent with previous studies [8,9]. This means that thicker corneas have smaller P/A ratios, which is likely due to the increased posterior curvature that is seen in thinner corneas [10]. This negative correlation persisted with postoperative P/A in PRK at 4 mm, 5 mm, and 6 mm. Interestingly, there was no correlation between pachymetry and P/A ratio after LASIK or SMILE at any diameter. This could suggest that despite reduction of corneal thickness after LASIK or SMILE, the P/A ratio remains relatively constant. Other studies have shown that thinner corneas following refractive surgery can result in posterior corneal steepening in the paracentral zone, which is consistent with our findings [11].

Anterior keratometry was found to negatively correlate with preoperative P/A in the 4-mm zone. This suggests that a smaller preoperative P/A is associated with greater anterior keratometric power, which is consistent with what is found in the literature [8]. Anterior keratometry was still negatively correlated with P/A postoperatively in PRK patients at 4 mm, 5 mm, and 6 mm. Interestingly, anterior keratometry became positively correlated with P/A postoperatively in LASIK patients at 4 mm, 5 mm, and 6 mm. One study by Diener et al. looked at postoperative P/A in patients who underwent Descemet’s membrane endothelial keratoplasty (DMEK) and found a significant decrease in mean P/A as well as decreased anterior Km (flattened), suggesting a positive correlation between the two [12]. Despite the difference between LASIK and DMEK, this finding supports the idea that a multitude of factors influence the P/A ratio and that its change cannot be explained solely by a single variable.

Posterior keratometry was found to positively correlate with preoperative P/A in all patients, which reflects what has been reported in the literature [8]. Posterior keratometry continues to positively correlate with postoperative P/A in all zones, but only in PRK and SMILE. Interestingly, there is no correlation of posterior keratometry with postoperative P/A in LASIK at any zone. This suggests that patients with greater posterior keratometric power will have a larger P/A ratio postoperatively, but only in PRK and SMILE. For patients undergoing LASIK, any postoperative P/A ratio is equally likely for any preoperative posterior keratometry value. This could suggest that keratometric changes induced to the anterior cornea in LASIK are accompanied by a proportional change in the posterior cornea.

Multivariate analyses were then run using the above preoperative characteristics to generate predictive models of postoperative PA. Across all multivariate analyses, preoperative spherical equivalent had the greatest standard coefficient in LASIK, SMILE, and PRK, indicating that it is the greatest predictor of postoperative P/A ratio across all surgeries. Preoperative P/A ratio was also consistently the second greatest predictor in all groups. When looking at the model for each zone within each surgery, we found that the 6-mm paracentral zone consistently had the highest coefficient of determination in all surgeries (LASIK adjusted R^2^: 0.957, PRK adjusted R^2^: 0.934, SMILE adjusted R^2^: 0.894).

Using the 6-mm model in Table 5 in LASIK patients, we found it was able to predict postoperative P/A ratio within ±0.01 for 92% of patients and ±0.02 for 100% of patients when using preoperative characteristics. For PRK, the 6-mm model predicted ±0.01 for 91% of patients and ±0.02 for 100% of patients. The 6-mm model for SMILE similarly predicted P/A ratio within ±0.01 for 93% of patients and ±0.02 for 100% of patients. The high predictability of the P/A ratio could be useful in post-refractive patients who want to undergo cataract surgery, because inaccuracy in the P/A ratio has been shown to contribute up to 3.69% of the error in IOL calculations [1]. Traditional IOL calculations assume a fixed P/A ratio of 0.82 that is typical of corneas that have not undergone surgery. Since refractive surgery changes the P/A ratio, finding a way to predict that change could be useful in improving the accuracy of cataract surgery.

There are several limitations in our study. First, our study is retrospective and thus could be subject to selection bias and information bias. Prospective studies are needed to further characterize the validity of preoperative characteristics in predicting postoperative P/A ratio. Given the lack of post-refractive P/A ratios in the literature, it was difficult to validate our study by comparison. Secondly, our sample sizes are not the same across all groups, namely with SMILE having a much smaller relative population. Additionally, Scheimpflug imaging has certain limitations, and its accuracy in measuring keratometry can be affected by factors such as tear film quality, corneal deposits, and corneal scarring. Furthermore, our study did not account for the natural correlation that occurs between the left and right eyes of a single patient. However, the intercorrelation between eyes would produce greater variance in the data, and thus significance would be more difficult to achieve. Our study primarily made inferences on correlations that were significant, and the overall conclusions would likely remain the same. The demographics were also not completely normalized between surgical groups with PRK having thinner corneas on average, and SMILE having more myopic patients with less average cylinder diopters. However, our study did not directly compare the correlations between groups as this was not the goal of this study. Furthermore, each refractive surgery attracts patients with particular preoperative characteristics because they may be ideal candidates for a particular procedure. Despite the strong predictive value of our model, further research is needed to explore the clinical repercussions of P/A ratio, with recent studies looking at its ability to add precision in IOL calculations.

P/A ratios have been well-studied in patients prior to undergoing refractive surgery, but not postoperatively. Our study showed that age, spherical equivalent, asphericity, pachymetry, and keratometry not only correlated preoperatively with P/A ratios, but also continued to correlate with P/A postoperatively. Additionally, spherical equivalent and preoperative P/A had the strongest correlations with postoperative PA, which was represented by their contribution to the predictive models. The confluence of independent contributions of preoperative characteristics allows for an extremely accurate, predictive model of postoperative P/A ratio.

## Figures and Tables

**Table 1:** Population baseline characteristics.

	All	LASIK	PRK	SMILE
No. of Eyes	393	164	183	46
No. of Patients	215	91	100	25
Male	116	55	50	11
Female	99 *	36	50	14
Age (y)	33.45 ± 6.37	33.75 ± 7.14	33.273 ± 5.7	33.087 ± 5.92
	(20 to 51)	(20 to 51)	(20 to 45)	(22 to 45)
Cylinder (D)	−0.759 ± 0.63	−0.825 ± 0.65	−0.793 ± 0.65	−0.388 ± 0.34
	(−5.75 to 0)	(−5.75 to 0)	(−4.25 to 0)	(−1.25 to 0)
MRSE (D)	−3.644 ± 1.7	−3.308 ± 1.82	−3.598 ± 1.46	−5.025 ± 1.45
	(−7.71 to −2.66)	(−7.61 to −0.25)	(−7.30 to −0.37)	(−7.71 to −2.66)
P/A 4 mm	0.826 ± 0.02	0.826 ± 0.02	0.827 ± 0.02	0.823 ± 0.01
	(0.784 to 0.910)	(0.787 to 0.873)	(0.784 to 0.910)	(0.787 to 0.844)
P/A 5 mm	0.822 ± 0.02	0.822 ± 0.02	0.823 ± 0.02	0.819 ± 0.01
	(0.781 to 0.888)	(0.785 to 0.888)	(0.781 to 0.86)	(0.789 to 0.836)
P/A 6 mm	0.821 ± 0.01	0.821 ± 0.01	0.822 ± 0.02	0.818 ± 0.01
	(0.781 to 0.863)	(0.785 to 0.863)	(0.781 to 0.86)	(0.789 to 0.833)
Pachymetry (µm)	536.784 ± 31.23	547.793 ± 25.28	523.53 ± 30.29	550.261 ± 33.03
	(453 to 617)	(501 to 617)	(453 to 593)	(489 to 615)
Anterior Q	−0.333 ± 0.12	−0.326 ± 0.13	−0.34 ± 0.12	−0.33 ± 0.11
	(−0.71 to 0.01)	(−0.71 to 0.01)	(−0.69 to 0.01)	(−0.60 to −0.14)
Posterior Q	−0.343 ± 0.14	−0.338 ± 0.14	−0.35 ± 0.14	−0.333 ± 0.12
	(−0.76 to 0.03)	(−0.73 to 0.03)	(−0.76 to −0.03)	(−0.52 to −0.12)
Keratometry (D)	43.901 ± 1.24	43.695 ± 1.29	44.101 ± 1.21	43.837 ± 1.03
	(40.00 to 47.80)	(40.00 to 47.20)	(41.20 to 47.80)	(41.70 to 46.00)

Notes: *—one female patient had a different surgery in each eye, MRSE—mean refracted spherical equivalent, P/A—posterior-to-anterior corneal curvature radii ratio, y—years, D—diopter, ±standard deviation, parentheses denote range.

**Table 2:** Correlations between preoperative characteristics with both preoperative and postoperative P/A ratios in LASIK, PRK, and SMILE.

	All (*n* = 393)		LASIK (*n* = 164)		PRK (*n* = 183)		SMILE (*n* = 46)		
Parameters	Preop P/A		Postop P/A		Postop P/A		Postop P/A		
	*r*	*p*	*r*	*p*	*r*	*p*	*r*	*p*	
Age	0.195 **	**<0.001**	0.115	0.141	0.051	0.496	0.131	0.386	4 mm
Sex	−0.007	0.886	0.060	0.447	0.121	0.103	−0.005	0.974	
Cylinder	0.011	0.821	0.037	0.634	0.055	0.458	0.096	0.525	
MRSE	−0.115 *	**0.023**	0.833 **	**<0.001**	0.791 **	**<0.001**	0.747 **	**<0.001**	
Anterior Q	0.031	0.544	0.200 *	**0.010**	0.180 *	**0.015**	−0.083	0.583	
Posterior Q	0.400 **	**<0.001**	0.277 **	**<0.001**	0.177 *	**0.017**	0.406 **	**0.005**	
Pachymetry	−0.450 **	**<0.001**	−0.131	0.094	−0.167 *	**0.024**	−0.169	0.262	
Anterior Km	−0.105 *	**0.038**	0.226 **	**0.004**	−0.225 **	**0.002**	−0.199	0.186	
Posterior Km	0.602 **	**<0.001**	−0.040	0.613	0.381 **	**<0.001**	0.440 **	**0.002**	
Preop P/A	1		0.289 **	**<0.001**	0.388 **	**<0.001**	0.573 **	**<0.001**	
Age	0.213 **	**<0.001**	0.133	0.091	0.072	0.335	0.167	0.267	5 mm
Sex	0.013	0.797	0.072	0.360	0.130	0.079	0.008	0.956	
Cylinder	−0.001	0.985	0.033	0.676	0.070	0.349	0.128	0.395	
MRSE	−0.106 *	**0.036**	0.837 **	**<0.001**	0.799 **	**<0.001**	0.772 **	**<0.001**	
Anterior Q	0.048	0.341	0.194 *	**0.013**	0.183 *	**0.013**	−0.076	0.617	
Posterior Q	0.376 **	**<0.001**	0.261 **	**<0.001**	0.155 *	**0.037**	0.374 *	**0.010**	
Pachymetry	−0.440 **	**<0.001**	−0.128	0.103	−0.161 *	**0.029**	−0.167	0.266	
Anterior Km	−0.092	0.069	0.224 **	**0.004**	−0.197 **	**0.008**	−0.204	0.175	
Posterior Km	0.580 **	**<0.001**	−0.062	0.431	0.369 **	**<0.001**	0.396 **	**0.006**	
Preop P/A	1		0.298 **	**<0.001**	0.391 **	**<0.001**	0.536 **	**<0.001**	
Age	0.239 **	**<0.001**	0.153 *	**0.050**	0.093	0.210	0.231	0.123	6 mm
Sex	0.017	0.731	0.079	0.317	0.144	0.052	0.022	0.887	
Cylinder	−0.006	0.912	0.038	0.628	0.095	0.202	0.148	0.325	
MRSE	−0.106 *	**0.036**	0.825 **	**<0.001**	0.779 **	**<0.001**	0.749 **	**<0.001**	
Anterior Q	0.064	0.208	0.197 *	**0.011**	0.186 *	**0.012**	−0.077	0.609	
Posterior Q	0.336 **	**<0.001**	0.251 **	**0.001**	0.131	0.077	0.351 *	**0.017**	
Pachymetry	−0.431 **	**<0.001**	−0.134	0.086	−0.160 *	**0.030**	−0.188	0.212	
Anterior Km	−0.077	0.128	0.240 **	**0.002**	−0.194 **	**0.009**	−0.219	0.144	
Posterior Km	0.556 **	**<0.001**	−0.070	0.371	0.379 **	**<0.001**	0.392 **	**0.007**	
Preop P/A	1		0.324 **	**<0.001**	0.410 **	**<0.001**	0.503 **	**<0.001**	

Notes: **bolded**—significant correlations, *—significance at *p* < 0.05, **—significance at *p* < 0.01, MRSE—mean refracted spherical equivalent, y—years, Km—keratometry, r—correlation coefficient, P/A—posterior-to-anterior corneal curvature radii ratio.

**Table 3:** Multivariate regression analysis of independent variables and postoperative P/A ratio in LASIK patients (*n* = 164).

4 mm				5 mm				6 mm			
	Partial ß	Std. ß	*p* Value		Partial ß	Std. ß	*p* Value		Partial ß	Std. ß	*p* Value
Intercept	−0.0304		0.54	Intercept	0.0088		0.861	Intercept	−0.0521		0.23
MRSE	0.0154	0.935	<0.001	MRSE	0.0152	0.942	<0.001	MRSE	0.0142	0.925	<0.001
Preop P/A	0.9048	0.468	<0.001	Preop P/A	0.8492	0.446	<0.001	Preop P/A	0.9512	0.478	<0.001
Posterior Km	−0.0156	−0.114	<0.001	Age	0.0005	0.128	<0.001	Age	0.0004	0.102	<0.001
Age	0.0004	0.086	<0.001	Posterior Km	−0.0150	−0.113	<0.001	Posterior Km	−0.0125	−0.096	<0.001
Anterior Q	0.0103	0.043	0.023					Anterior Q	0.0087	0.039	0.021
**Adjusted R^2^: 0.944**				**Adjusted R^2^: 0.941**				**Adjusted R^2^: 0.956**			

Notes: Partial ß—partial regression coefficient, Std. ß—standard regression coefficient, MRSE—mean refracted spherical equivalent, P/A—posterior-to-anterior corneal curvature radii ratio.

**Table 4:** Multivariate regression analysis of independent variables and postoperative P/A ratio in PRK patients (*n* = 183).

4 mm				5 mm				6 mm			
	Partial ß	Std. ß	*p* Value		Partial ß	Std. ß	*p* Value		Partial ß	Std. ß	*p* Value
Intercept	0.2887		<0.001	Intercept	0.2136		<0.001	Intercept	0.1675		<0.001
MRSE	0.0136	0.816	<0.001	MRSE	0.0135	0.831	<0.001	MRSE	0.0128	0.821	<0.001
Preop P/A	0.6831	0.476	<0.001	Preop P/A	0.7547	0.493	<0.001	Preop P/A	0.8084	0.526	<0.001
Anterior Q	0.0203	0.098	<0.001	Anterior Q	0.0172	0.086	<0.001	Anterior Q	0.0154	0.080	<0.001
Pachymetry	−5.84 × 10^−5^	−0.073	0.007	Age	0.0003	0.060	0.003	Age	0.0003	0.066	<0.001
				Pachymetry	−4.72 × 10^−5^	−0.060	0.008	Pachymetry	−4.41 × 10^−5^	−0.059	0.007
**Adjusted R^2^: 0.900**				**Adjusted R^2^: 0.930**				**Adjusted R^2^: 0.934**			

Notes: Partial ß—partial regression coefficient, Std. ß—standard regression coefficient, MRSE—mean refracted spherical equivalent, P/A—posterior-to-anterior corneal curvature radii ratio.

**Table 5:** Multivariate regression analysis of independent variables and postoperative P/A ratio in SMILE patients (*n* = 46).

4 mm				5 mm				6 mm			
	Partial ß	Std. ß	*p* Value		Partial ß	Std. ß	*p* Value		Partial ß	Std. ß	*p* Value
Intercept	0.2756		0.001	Intercept	0.3848		<0.001	Intercept	0.3815		<0.001
MRSE	0.0103	0.680	<0.001	MRSE	0.0108	0.762	<0.001	MRSE	0.0100	0.774	<0.001
Preop P/A	0.6570	0.454	<0.001	Preop P/A	0.5001	0.343	<0.001	Preop P/A	0.4972	0.349	<0.001
Post. Q	0.0474	0.291	<0.001	Post. Q	0.0421	0.278	<0.001	Post. Q	0.0349	0.251	<0.001
				Age	0.0006	0.181	0.008	Age	0.0006	0.217	0.002
**Adjusted R^2^: 0.873**				**Adjusted R^2^: 0.890**				**Adjusted R^2^: 0.894**			

Notes: Partial ß—partial regression coefficient, Std. ß—standard regression coefficient, MRSE—mean refracted spherical equivalent, P/A—posterior-to-anterior corneal curvature radii ratio, Post. Q—posterior Q value.

## Data Availability

Data in this study are available on request from corresponding author.
